# Changes in Gastric Smooth Muscle Cell Contraction during Pregnancy: Effect of Estrogen

**DOI:** 10.1155/2019/4302309

**Published:** 2019-04-04

**Authors:** Othman A. Al-Shboul, Hanan J. Al-Rshoud, Ahmed N. Al-Dwairi, Mohammad A. Alqudah, Mahmoud A. Alfaqih, Ayman G. Mustafa, Mohammad Jaafar

**Affiliations:** ^1^Department of Physiology and Biochemistry, Faculty of Medicine, Jordan University of Science and Technology, Irbid 22110, Jordan; ^2^Department of Anatomy, Faculty of Medicine, Jordan University of Science and Technology, Irbid 22110, Jordan; ^3^Faculty of Medicine, Al-Balqa Applied University, Salt 19117, Jordan

## Abstract

It is well known that pregnancy is associated with frequent gastrointestinal (GI) disorders and symptoms. Moreover, previous reports have shown that estrogen, which changes in levels during pregnancy, participates in the regulation of GI motility and is involved in the pathogenesis of various functional disorders in the stomach. The aim of the current study was to explore the changes in the expression of estrogen receptors (ERs) and examine the effect of estrogen on nitric oxide- (NO-) cyclic guanosine monophosphate (cGMP) pathway and thus relaxation in gastric smooth muscle cells (GSMC) during pregnancy. Single GSMC from early-pregnant and late-pregnant Sprague-Dawley rats were used. Protein and mRNA expression levels of ERs were measured via specifically designed enzyme-linked immunosorbent assay (ELISA) and polymerase chain reaction (PCR), respectively. NO and cGMP levels were measured via specifically designed ELISA kits. Effect of estrogen on acetylcholine- (ACh-) induced contraction of single GSMC was measured via scanning micrometry in the presence or absence of the NO synthase inhibitor,* N*-nitro-L-arginine (L-NNA), or guanylyl cyclase inhibitor, 1H-[1,2,4]oxadiazolo[4,3,-a]quinoxalin-1-one (ODQ). Estrogen increased both NO and cGMP levels and their levels were greater in early compared to late pregnancy. Expression of ERs was greater in early compared to late pregnancy. ACh induced greater contraction of GSMC in late pregnancy compared to early pregnancy. Estrogen inhibited ACh-induced contraction in both periods of pregnancy. Importantly, pretreatment of GSMC with either L-NNA or ODQ abolished estrogen inhibitory action on muscle contraction. In conclusion, GSMC contractile behavior undergoes drastic changes in response to estrogen during pregnancy and this might explain some of the pregnancy-associated gastric disorders.

## 1. Introduction

Various GI tract disorders are commonly reported and encountered problems in normal pregnancy and constitute one of the most frequent symptoms during gestation [1]. For example, pregnancy is usually associated with nausea, vomiting, various degrees of stomach emptying disorders, and varying degrees of constipation resulting from reduced colonic contractile activity [2–4]. In support of the interrupted GI smooth muscle myoelectric and motor behavior during pregnancy, previous research in pregnant women reported a lowered gallbladder contractile activity [5, 6] and esophageal sphincter pressure [7–9], a delayed gastric emptying [10, 11], and reduced small intestinal [12] and colonic transit [13].

Pregnancy is characterized by raised levels of circulating steroid hormones, namely, estrogens and progesterone, which increase with advancing gestational age [14, 15]. These hormones play central roles in maintenance of pregnancy and initiation of parturition by modulating myometrial contractility and excitability. In addition, recent studies have shown that estrogen and progesterone target other smooth muscle-made body organs, besides myometrium, such as GI tract, bladder, and blood vessels [16].

Estrogen regulates gene expression in estrogen-targeted tissues through binding one of two members of the superfamily of steroid hormone nuclear receptors, the estrogen receptor *α* (ER*α*) and the estrogen receptor *β* (ER*β*) [17]. These two subtypes of ERs are coded by separate genes [18, 19]. They are expressed alone or together in several different tissues of the body including the female reproductive tract, the vasculature, and the GI tract [19–21]. Estrogen was also found to induce some rapid signaling events or nongenomic events in a variety of cell types giving strong functional evidence that surface membrane ERs (known as GPER) are also involved in the rapid relaxing effects of estrogen [22].

Estrogen was reported to produce relaxation in smooth muscles of gall bladder [23], trachea [24], urinary bladder [25], blood vessels [26], and colon [27, 28]. In addition, estrogen induces relaxation of vascular smooth muscle by a process that involves activation of NO-cGMP pathway [29]. Most importantly, we recently reported estrogen- and progesterone-induced relaxation in stomach smooth muscle cells via activation of the NO-cGMP pathway [30, 31].

Smooth muscle is physiologically a key player in developing and maintaining GI tract normal motility behavior. Smooth muscle undergoes contraction and relaxation by targeting phosphorylation and dephosphorylation of the 20-kDa regulatory myosin light chain (MLC_20_), respectively [32, 33]. Cyclic adenosine monophosphate (cAMP) and cyclic guanosine monophosphate (cGMP) are the key molecules produced in GSMC by most relaxing agents [33]. These relaxing molecules activate two important downstream kinases, cAMP-activated protein kinase A (PKA) and cGMP-activated protein kinase G (PKG), which induce relaxation in smooth muscle by targeting various proteins and enzymes in relaxation pathway of GSMC [34]. Nitric oxide (NO) generated by the gut smooth muscle-expressed nitric oxide synthase (NOS) induces the production of cGMP by activating the soluble guanylyl cyclase (sGC) [33, 35, 36]. Cyclic nucleotide elimination pathways include degradation by phosphodiesterases (PDEs) and active export into the extracellular space via members of the multidrug resistance protein (MRP) family (also known as the ATP-binding cassette transporter family) [37].

In this study, we test the hypothesis that there is a change in contraction of stomach smooth muscle cells and estrogen-induced effect on muscle contractile behavior during pregnancy which may be a result of differences in the expression and/or activity of ER subtypes. We also sought to explore the change in estrogen effect on NO/cGMP pathway in the stomach muscle cells during pregnancy. This research in pregnant GMSC is in continuation of our previously reported effect of estrogen and progesterone in nonpregnant female [30, 31]. Our findings may be of considerable importance in understanding the cause of the various pregnancy-associated GI tract motility disorders and would further pave the way for understanding the ER-mediated smooth muscle contraction-relaxation pathways and thereby establishing novel therapeutic approaches for treatment of GI disorders.

## 2. Materials and Methods

### 2.1. Animals

The Animal Care and Use Committee at Jordan University of Science and Technology approved all the procedures conducted. The animal house of the Jordan University of Science and Technology provided and housed the animals under standardized conditions (temperature 20-22°C, humidity 50-60%, and 12 h light/dark cycle) and allowed free access to food and tap water throughout the experiments. Female virgin rats were mated with male Sprague-Dawley rats. Day 1 of gestation was designated as the day a vaginal plug was observed. 17 early-pregnant (gestation day 10) and 16 late-pregnant (gestation day 20) rats were used in the study.

### 2.2. Isolation of Stomach Smooth Muscle Cells

Rats were euthanized by CO_2_ inhalation for at least 5 min. Euthanasia was confirmed by incising the diaphragm with a scalpel blade. The stomach was immediately excised following euthanasia. Smooth muscle cells were isolated from the stomachs of pregnant female Sprague-Dawley rats (12 weeks of age, 250-300 g) by sequential enzymatic digestion, filtration, and centrifugation as described previously [38, 39]. Briefly, muscle strips from the stomach were dissected and incubated at 31°C for 30 min in HEPES buffer composed of 120 mM NaCl, 4 mM KCl, 2.0 mM CaCl_2_, 2.6 mM KH_2_PO_4_, 0.6 mM MgCl_2_, 25 mM HEPES (4-(2-hydroxyethyl)-1-piperazineethanesulfonic acid), 14 mM glucose, 2.1% Eagle's essential amino acid mixture, 0.1% collagenase, and 0.01% soybean trypsin inhibitor (pH was adjusted to 7.4). The partly digested strips were washed twice with 50 ml of enzyme-free medium and the muscle cells were allowed then to disperse spontaneously for 30 min. Filtration through 500 *μ*m Nitex mesh was used to harvest the cells followed by centrifugation twice at 350 g for 10 min to eliminate broken cells and organelles. Experiments were done within 2–3 h of cell collection.

### 2.3. Protein Expression of Estrogen Receptors (ER*α* and ER*β*) via ELISA

GSMCs collected from 10 ml muscle cell suspension (3x10^6^ cells/ml) were centrifuged (20,000 x g at 4°C for 1 min) and the pellet was snap-frozen in liquid nitrogen and homogenized using a Teflon glass pestle in 400 *μ*l ice-cold distilled water. Following the centrifugation of the lysates at 20,000 x g at 4°C for 10 min, the protein concentration in the supernatant was determined with a Dc protein assay kit (Bio-Rad Laboratories, Inc.). Samples containing equal amounts of protein were used for quantification of ER*α* and ER*β* using the ELISA kits according to the manufacturer's protocol.

### 2.4. Messenger RNA Expression of Estrogen Receptors (ER*α* and ER*β*) by Real-Time PCR (qRT-PCR)

Real-time PCR was performed on cDNA samples synthesized from total RNA isolated from GSMC using Quick-RNA Mini prep kit from Zymo Research (Genesee Scientific, San Diego, USA) and treated with RNase-free DNase I. Spectrophotometric analysis of RNA measured at OD260/OD280 ratios were between 1.8 and 2.2 and the integrity was checked by gel electrophoresis. A total of 500ng/ul RNA was converted into cDNA for real-time PCR assay using Prime Script RT reagent Kit (Takara; Clontech). Real-time PCR for each primer was performed using KAPA SYBR® FAST qPCR Master Mix (2X) Kit via the StepOne Real-Time PCR System (Applied Biosystems, Foster City, CA, USA). The following time and temperature profile was used for the real-time PCR reactions: 95°C for 3 min; 40 cycles of a series consisting of 3 s at 95°C, 30 s at 60°C, and 30 s at 72°C. The optimal annealing temperatures were determined empirically for each primer set. All reactions were performed in triplicate and results were analyzed with Rotor-Gene Q software (Qiagen). All results were normalized to GAPDH control and used for analysis. The sequences of specific primers for ER*α* were forward, 5′-GCTTTTGAACCAGCAGGGTGGC-3′ and reverse, 5′-AACAAGGCCATTCCCGAGGC-3′, for ER*β* was forward, 5′-GGATGGAGGTGCTAATGGTGGG-3′ and reverse, 5′- CACTTCCCCTCATCCCTGTCCA-3′, and for GAPDH (internal control) was forward, 5′- TGGTGGACCTCATGGCCTAC-3′ and reverse 5′-CAGCAACTGAGGGCCTCTCT-3′.

### 2.5. Measurement of Contraction in Dispersed GSMC

Scanning micrometry was used to measure contraction of isolated muscle cells as described previously [39, 40]. An aliquot of muscle cells (0.4 mL containing 10^4^ cell/mL) was treated with estrogen (1 *μ*M), estrogen and ODQ (guanylyl cyclase inhibitor) (1 *μ*M), or estrogen and L-NNA (NO synthase inhibitor) (1 *μ*M) for 10 min and then with acetylcholine (ACh) (0.1 *μ*M) for another 10 min. Acrolein (0.1% final concentration) was used to terminate the reaction and a drop of cell suspension was placed on a slide under a cover slip and viewed using an inverted Nikon TMS-f microscope (Nikon, Tokyo, Japan). Cell images were acquired using a Canon digital camera (Canon Inc., Tokyo, Japan) and ImageJ acquisition software (version 1.45s; National Institutes of Health, Bethesda, MA, USA). Cell length in the absence of any treatment was taken as resting cell length. Contraction was expressed as the percentage decrease of mean cell length of 30 muscle cells as compared with resting cell length.

### 2.6. Measurement of Smooth Muscle NO

Nitric oxide (NO^2−^/NO^3−^) assay kit was utilized to indirectly measure NO concentration in smooth muscle samples by determining both nitrate and nitrite levels according to the manufacturer's instructions.

### 2.7. Measurement of Smooth Muscle cGMP

Cyclic GMP level was measured in smooth muscle samples using ELISA kit according to the manufacturer's instructions.

### 2.8. Materials

A DC protein assay kit (cat. no. 500-0116) for measuring protein concentration was obtained from Bio-Rad Laboratories, Inc., Hercules, CA, USA. Cyclic GMP colorimetric ELISA kit (cat. no. STA-505) was obtained from Cell BioLabs, Inc., San Diego, CA, USA. Nitric oxide (NO^2−^/NO^3−^) assay kit (cat. no. 23479 Sigma) was obtained from Sigma-Aldrich (Merck KGaA, Darmstadt, Germany). 1H-[1,2,4]Oxadiazolo[4,3-a]quinoxalin-1-one (ODQ; cat. no. ab120022) and* Nω*-nitro-L-arginine (L-NNA; cat. no. ab141312) were obtained from Abcam (Cambridge, UK). A 500 *μ*m Nitex mesh was purchased from Sefar AG, Heiden, Switzerland. All remaining chemicals were from Sigma-Aldrich (Merck KGaA, Darmstadt, Germany). Stock solution of estrogen was prepared in 100% ethanol. Stock solutions of ODQ and L-NNA were prepared in dimethylsulfoxide (DMSO). The final concentration of ethanol and DMSO used was 1% (volume/volume).

### 2.9. Statistical Analysis

Results are expressed as the mean ± standard error of the mean (SEM). Statistical analysis of all experiments was performed using Prism 5.0 software (GraphPad Software, San Diego, CA). Statistical differences between two means were determined by Student's* t*-test. Statistical differences between multiple groups were tested using the one-way analysis of variance followed by Tukey's post hoc test. Differences were considered significant at* P *< 0.05.

## 3. Results

### 3.1. ER Expression

**Figure 1:**
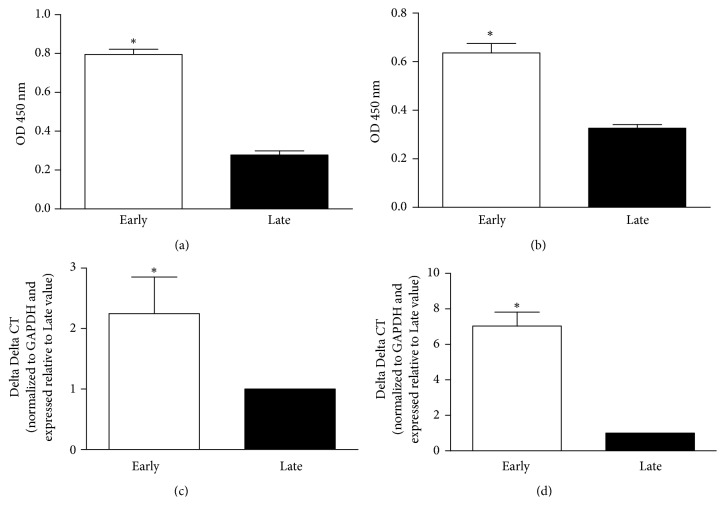
Expression of ER*α* and ER*β* in GSMC from early- and late-pregnant rats. (a) and (b) Representative protein expression levels of ER*α* and Er*β*, respectively, by ELISA. Protein expression level is expressed as OD 450 nm. ER*α* and ER*β* proteins were more expressed in GSMCs from early-pregnant rats compared with those in GSMCs from late-pregnant rats. (c) and (d) Messenger RNA levels of ER*α* and ER*β* were measured by qRT-PCR in RNA isolated from the GSMC of early- and late-pregnant animals and expressed as delta delta CT normalized to GAPDH and relative to level in late-pregnant sample. ER*α* and ER*β* mRNAs were more expressed in GSMCs from early-pregnant rats compared with those in GSMCs from late-pregnant rats. Values shown are representative of three independent experiments performed in triplicate.*∗P* < 0.05 measurements in early-pregnant rats are significantly different from corresponding measurements in late-pregnant ones.

ELISA analysis revealed greater protein amount of both ER*α* (2.86 folds) and ER*β* (1.94 folds) (*P* < 0.05) in the early-pregnant GSMC compared to late-pregnant cells (Figures 1(a) and 1(b)). Parallel, real-time PCR showed greater mRNA expression of both ER isoforms (*P* < 0.05) in the early-pregnant GSMC compared to late-pregnant cells (Figures 1(c) and 1(d)).

### 3.2. Changes in Estrogen-Induced NO Formation

**Figure 2:**
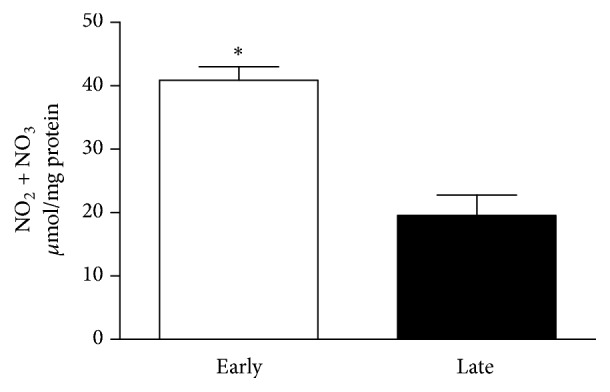
Estrogen-induced production of nitric oxide (NO) in GSMC from early- and late-pregnant rats. Total NO metabolites (nitrate + nitrite) were measured as indicators for nitric oxide (NO) levels. Treatment of GSMC with estrogen (E2) significantly increased NO levels in GSMC. Values shown are representative of three independent experiments performed in triplicate. Samples were collected from 6 early-pregnant and 7 late-pregnant rats. *∗* measurements in early-pregnant rats are significantly different (*P *< 0.05) from corresponding measurements in late-pregnant rats.

Treatment of muscle cells with estrogen significantly increased NO levels above basal levels in both groups of cells (*P* < 0.05). Importantly, estrogen-induced NO production was higher in cells from early-pregnant animals compared to cells from late-pregnant animals (2.09 folds) (*P* < 0.05) (Figure 2).

### 3.3. Changes in Estrogen-Induced cGMP-Formation

**Figure 3:**
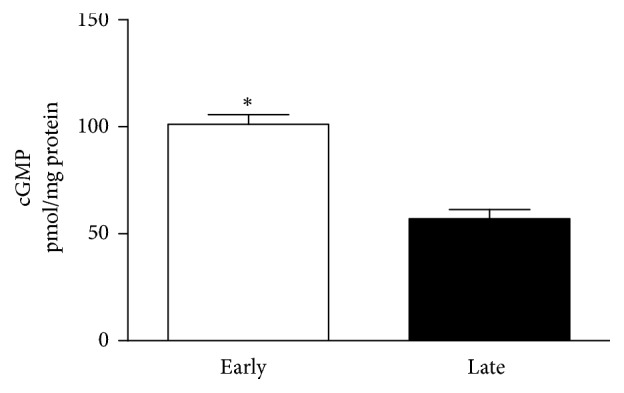
Estrogen-induced production of cyclic guanosine monophosphate (cGMP) in GSMC from early- and late-pregnant rats. Incubation with estrogen (E2) significantly increased cGMP levels in GSMC. Values shown are representative of three experiments performed in triplicate. Samples were collected from 6 early-pregnant and 7 late-pregnant rats. *∗* measurements in early-pregnant rats are significantly different (*P* < 0.05) from corresponding measurements in late-pregnant rats.

Treatment of GSMC with estrogen significantly increased cGMP levels above basal levels in both groups of cells (*P* < 0.05). Importantly, estrogen increased cGMP to higher levels in early-pregnant cells compared to late-pregnant cells (1.77 folds) (*P* < 0.05) (Figure 3).

### 3.4. Changes in Estrogen Effect on Muscle Cell Contraction

**Figure 4:**
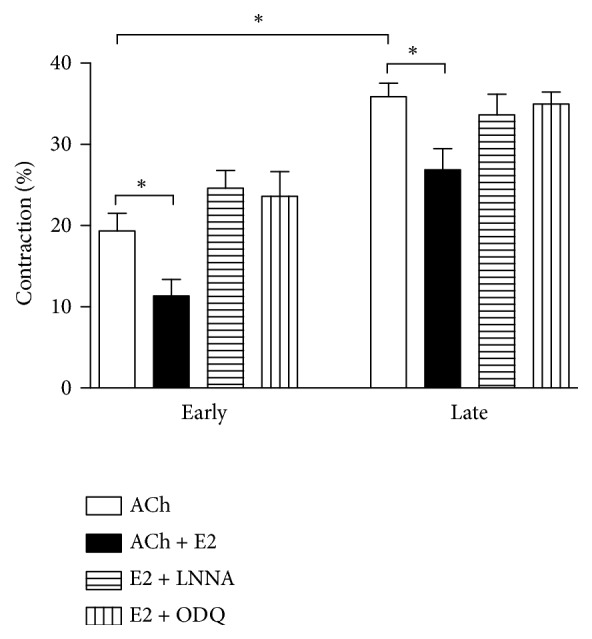
Changes in contraction of GSMC from early- and late-pregnant rats. Contraction of GSMC in response to ACh was significantly greater in cells from late-pregnant animals compared to cells from early-pregnant animals. Estrogen (E2) significantly inhibited ACh-induced contraction in GSMC of both groups of cells. Importantly, estrogen inhibition of contraction was greater in cells from the early-pregnant animals (~41% reduction) compared to cells from the late-pregnant animals (~25% reduction). L-NNA (NO synthase inhibitor) and ODQ (guanylyl cyclase inhibitor) significantly blocked estrogen effect in both groups. (*∗P *< 0.05 for the groups in comparison, n = 30 cells from 10 different rats).

Resting muscle length (i.e., the length of cells in the control group not treated with ACh) was indifferent in both early- and late-pregnant cells. ACh elicited muscle cell contraction in both pregnancy groups. However, contraction in response to ACh was significantly greater in cells from late-pregnant animals compared to cells from early-pregnant ones (*P* < 0.05). Importantly, pretreatment of gastric muscle cells with estrogen significantly inhibited ACh-induced contraction in cells of both early- and late-pregnant rats (*P* < 0.05). Notably, estrogen-induced relaxation was greater in cells from the early-pregnant animals (~41% reduction) compared to cells from the late-pregnant animals (~25% reduction) (*P* < 0.05) (Figure 4).

### 3.5. Effect of the Blockade of NO Synthase and sGC on Estrogen-Induced Relaxation

We next targeted exploring the effect of NO synthase blocker (L-NNA) and sGC blocker (ODQ) on estrogen-induced inhibition of muscle contraction of GSMC from both pregnancy groups. Both L-NNA and ODQ significantly reduced the estrogen-induced inhibition of muscle contraction in muscle cells from both early- and late-pregnant rats (*P* < 0.05) (Figure 4).

## 4. Discussion

We report in this study greater expression of ER isoforms (ER*α* and ER*β*) and decreased contraction in gastric muscle cells from early-pregnant rats compared to cells from late-pregnant rats. This estrogen-induced greater gastric muscle cell relaxation in early-pregnant animals is mediated via greater release of NO and higher production of cGMP.

GI tract disorders are one of the most common symptoms in pregnancy. The factors regulating GI function during pregnancy are poorly understood. Understanding of these processes at cellular and molecular levels is essential for development of new therapeutic strategies. We previously found that estrogen affects gastric smooth muscle cell contractility and induces relaxation [30]. Because the hormone profiles change in different phases of pregnancy, we elected to study animals in early and late pregnancy.

High levels of estrogen in the serum of pregnant female suggest that estrogen may have an essential role in regulating gastric muscle gene expression and thus contractile behavior during pregnancy, similar to its effect on myometrial gene expression and contractility [41, 42]. Indeed, few studies have examined expression of ER in the GI tract. Previous northern analysis showed ER expression in the rat upper GI tract [43]. Both ER*α* and ER*β* mRNA were detected in the epithelium of the stomach and upper intestine by RT-PCR [44]. In the midgestational human fetus, ER*α* and ER*β* mRNAs were coexpressed in stomach and colon with lower levels in small intestine, as determined using RT-PCR [45].

We aimed first to explore differences in ER gene expression in GSMC during the two phases of pregnancy. Interestingly, we found for the first time that GSMC express greater ER, of both isoforms, in early pregnancy compared to late pregnancy. Greater uterine expression of ER is believed to help in preparation of the uterus for implantation and decidualization during early pregnancy [46]. In the myometrium, and parallel to our stomach findings, Geimonen et al. examined the levels of ER in term samples and were found to be very low [47]. Similarly, rat placenta was found to express both isoforms of ER and importantly their levels of expression decrease at the end of pregnancy near parturition stage [48]. Our ER expression results suggest a similar expression pattern in the stomach. Previous research has shown that trophoblast production of interferon tau, the pregnancy recognition hormone, during the late pregnancy acts in a paracrine fashion to suppress endometrial transcription of ER genes [49, 50]. Whether this mechanism exists in the stomach is not known yet and needs to be further explored.

Our study shows an increased contraction of stomach smooth muscle cells from late-pregnant animals in response to acetylcholine, M3 receptor agonist [51], compared to cells from early-pregnant animals. Most importantly, estrogen induced relaxation in GSMC and the magnitude of this relaxation was greater in early-pregnant rats compared to late ones. Increased expression of ER might explain this different estrogen effect and might contribute to the disrupted emptying function of the stomach reported in previous studies [52]. However, future studies using specific ER blockers will confirm the role of ER, if any, in such action.

The relaxation induced by estrogen was caused by NO production generated by stimulation of NO synthase as this estrogen-induced relaxation was abolished in the presence of the NO synthase inhibitor,* N*-nitro-L-arginine (L-NNA). We recently confirmed the production of NO by estrogen in female gastric smooth muscle cells [30]. Because we used freshly dispersed GSMC (to avoid the contribution of other players of the multicellular stomach compartment), NO production is via the previously identified smooth muscle NO synthase (NOS) [35]. The mechanism by which NO causes relaxation of the GSMC is mostly the stimulation of soluble guanylate cyclase resulting in cGMP formation [30]. Blockade of the estrogen-induced relaxation by guanylyl cyclase inhibitor, 1H-[1,2,4]oxadiazolo[4,3,-a]quinoxalin-1-one (ODQ), supports a role for the NO-cGMP pathway. Greater estrogen relaxation and more activation of NO-cGMP pathway in the stomach muscle cells from the early-pregnant animals compared to cells from the late-pregnant animals might be related to the higher expression of ER. Repeating these experiment using specific ER blockers might unravel such possibility. The NO-cGMP relaxation pathway is present in the human uterus and may be responsible for maintaining uterine quiescence during pregnancy [53]. A decreased responsiveness in uterine relaxation to nitric oxide at term may play a role in the initiation of labor [54, 55]. Our findings strongly propose similar molecular mechanism in the stomach during pregnancy.

The measurement of* in vitro* tonic contractility is one weakness of this study as phasic contractility that occurs normally* in vivo* should be measured in future studies taking into consideration the real integrated participation of various cell types and players in gastric motor activity such as wall muscle, enteric and extrinsic nervous system, interstitial cells of Cajal, and various hormonal factors. Additionally, only fixed doses of drugs were used in the current research. So repeating these experiments by establishing a dose-response curve would much strengthen our findings.

The rat as an animal model has been used due to the sensitivity of its GI tract to steroidal hormones as observed in GI changes during pregnancy which are similar to humans. Yet observations must be interpreted with care, as the rat as a consistent species avoids the differences related to age, body weight, ethnic background, comorbidities, and other confounding factors that are often encountered in human studies. Performing similar studies on human gastric muscle cells would further enhance our understanding of the mechanisms of stomach motility disorders associated with pregnancy.

## 5. Conclusions

Expression of the two isoforms of ERs (*α* and *β*) is decreasing with advancing pregnancy and this is associated with increased stomach muscle cell contraction. Estrogen induces gastric smooth muscle cell relaxation that is greater in the early period of pregnancy that is caused by enhanced stimulation of the NO-cGMP pathway. Further understanding of the changes in stomach contractile behavior during pregnancy will better characterize pregnancy-associated GI disorders and will enable more effective treatment for such disturbances.

## Data Availability

The data used to support the findings of this study are available from the corresponding author upon request.
